# The Expression of Cyclooxygenase-2 in Cervical Intraepithelial Neoplasia and Cervical Cancer

**DOI:** 10.7759/cureus.66473

**Published:** 2024-08-08

**Authors:** Nuzhath Shaik, Savitri M Nerune, Vijayalaxmi S Patil, Sneha Jawalkar

**Affiliations:** 1 Pathology and Laboratory Medicine, Al-Ameen Medical College, Vijayapura, IND; 2 Pathology and Laboratory Medicine, Shri BM Patil Medical College, Hospital and Research Center, Bijapur Liberal District Education (BLDE) Deemed to be University, Vijayapura, IND; 3 Pathology, Shri BM Patil Medical College, Hospital and Research Center, Bijapur Liberal District Education (BLDE) Deemed to be University, Vijayapura, IND

**Keywords:** cycloxygenase-2, cervical neoplasia, overexpression, immunohistochemistry, cox-2, cervical cancer

## Abstract

Aim

To examine the relationship between tumor differentiation, parametrial, and lymphovascular invasion, as well as the differential expression pattern of cyclooxygenase-2 (COX-2) in cervical intraepithelial neoplasia and various forms of cervical cancer.

Methods

Histologically diagnosed cases of in-situ and malignant lesions of the cervix were included in the study. Two sections were cut from paraffin blocks. One section was stained with Haematoxylin and Eosin (H&E) for morphologic diagnosis, and the other sections were subjected to COX-2 immunohistochemical staining. Cases of colon carcinoma were taken as positive controls. Cytoplasmic and membrane staining of tumor cells were considered as positive staining, and grading was done.

Results

Out of the 62 patients, 40 cases (64.5%) showed positive expression of COX-2 in squamous cell carcinoma when compared to in-situ cervical intraepithelial neoplasia and adenocarcinoma. The results were statistically significant, with a p-value of 0.003.

Conclusion

COX-2 expression is directly proportional to the level of grading of the tumor. The higher the grading, the higher the expression of COX-2. Selective COX-2 inhibitors increase the efficacy of chemotherapy or radiotherapy.

## Introduction

The second most common cause of death in women worldwide is cervical cancer, and it is a major cause of death in the developing world. In developed countries such as the United States, in the year 2000, 12,800 new cases of cervical cancer and 4600 deaths were reported. It is the most prevalent issue in developing nations, and worldwide, nearly 500,000 new cases of cervical cancer are discovered each year [1].

Cyclooxygenase (COX)-1 and 2 are two isoenzymes that, in the presence of free arachidonic acid, synthesize prostaglandin, which is an active lipid compound. Along with the inflammatory activity, prostaglandins are also involved in the growth of the tumor and even in carcinogenesis [2].

COX-1 and COX-2 have different properties, both morphologically as well as biologically. COX-1 is a housekeeping gene, whereas COX-2 is an immediate-early response gene. COX-2 is induced by growth factors, tumor promoters, oncogenes, and also by carcinogens [1]. By mediating pathologic processes that affect mitogenesis, cellular adhesions, and even immune surveillance, COX-2 promotes the proliferation, growth, and spread of the tumor. In transformed cells and malignant tissues, COX-2 is overexpressed. Few studies show inhibition of apoptosis and tumor angiogenesis stimulation by COX-2. Selective COX-2 inhibitors increase the efficacy of chemotherapy or radiotherapy in the treatment of human cancer.

Infection with human papillomavirus is the main cause of carcinoma of the cervix. Viral oncogenic proteins (E5, E6, and E7) increase the expression of COX-2 by activating the COX-2/prostaglandin E2 pathway, and it could be a significant prognostic marker for uterine cervical carcinomas [1-3].

During the early stages of cell replication or differentiation, COX-2 is present within the cells. In high-grade squamous cell carcinoma of the esophagus, adenomatous and metaplastic lesions of the stomach, pre-neoplastic lesions of the lung, pre-invasive neoplasias of the bladder, pancreas, as well as breast, expression of COX-2 is increased [4,5]. In light of the paucity of research on the expression of COX-2 in cervical cancer, we have examined the expression pattern of COX-2 in Cervical Intraepithelial Neoplasia (CIN), various forms of cervical cancer and its relationship to tumor differentiation, parametrial and lymphovascular invasion in the present study [6].

## Materials and methods

This study, which was prospective in nature, was conducted from January 2020 to July 2022 on all specimens from hysterectomy and cervical biopsies that were received by the pathology department's histopathology section. All the histologically diagnosed cases of in-situ and malignant lesions of the cervix were included and the patients who were on treatment with chemotherapy or radiotherapy prior to surgery were excluded from the study.

Methods of collection of data

Paraffin blocks were acquired from the Histopathology Section of the Department of Pathology in instances of cervical carcinoma that were histologically confirmed, including adenocarcinoma, squamous cell carcinoma, and in-situ cases. The time frame for our study was from January 1, 2020, to July 31, 2022. If imaging results were available, they were reported together with full clinical data.

The tissues underwent normal treatments after being fixed in 10% buffered formalin. Three sections, each measuring four microns in thickness, were cut from each tissue block. Hematoxylin and Eosin (H&E) staining was applied to one of these sections in order to analyze its morphology. Important variables were evaluated, including lymphovascular invasion, invasion depth, and histologic grade. The condition of the lymph nodes in hysterectomy specimens was also assessed.

After being coated with poly-L-lysine, two sections were put on slides and stained with cycloxygenase-2 (COX-2) immunohistochemically. A supersensitive polymer-based detection system (BioGenex, Fremont, California) and a rabbit monoclonal antibody for COX-2 were employed in this procedure. After being embedded in paraffin and fixed in formalin, tissue sections were deparaffinized and rehydrated using a series of alcohol and water solutions in order to prepare them for staining. In a microwave oven, heat treatment was used to retrieve antigens. Tris-buffered saline (TBS) was used to wash the sections, and then they underwent a 10-minute power block and a 10-minute peroxide blocking step. They were then treated for an hour at 4°C with the primary antibody. The slides were cleaned and rinsed again for 30 minutes with TBS before being added to and then incubated for a duration of 30 minutes, during which time they were treated with a secondary antibody.

Diaminobenzidine (DAB) was applied and allowed to incubate for ten minutes after being cleaned in the buffer. After that, the slides were cleaned and given a hematoxylin counterstain. Cases of colon cancer were used as positive controls. The tumor cells' cytoplasmic and membrane staining was indicative of positive staining.

Undetected, low (expressed in 10% of tumor cells), moderate (10-50% positive tumor cells), and high (>50% positive tumor cells) were the classifications for positive staining patterns. Fisher's two-tailed exact test with the Freeman-Halton extension was used for statistical analysis. The expression patterns were classified as expressed (low, moderate, and high positive) and not expressed (including undiagnosed cases) for statistical purposes.

Statistical analysis

The Statistical Package for the Social Sciences (SPSS) version 20 (IBM Inc., Armonk, New York) was used to perform statistical analysis on the data which was collected and placed into a Microsoft Excel sheet (Microsoft, Redmond, Washington). An independent t-test was used to compare continuous variables with normally distributed distributions. For non-normally distributed variables, the Mann-Whitney U test was employed. The chi-square test was used to compare categorical variables. For quantitative data, correlation and scatter diagrams were used to examine relationships between variables; for qualitative data, the chi-square test was employed. A p-value of less than 0.05 was deemed statistically significant.

## Results

Fourteen hysterectomy specimens and 48 cervical biopsies were included among the 62 cases. With a mean age of 54.61 years, the patients' age ranged from 22 to 90 years. The correlation of COX-2 expression in cervical carcinoma performed using the Pearson Chi-squared test and likelihood ratio calculation is shown in Table 1.

**Table 1:** Correlation of COX-2 expression in cervical carcinoma performed using the Pearson Chi-squared test and likelihood ratio calculation Pearson Chi-squared p-value: 0.003 (significant); Likelihood ratio p-value: 0.001 (significant); Df - degrees of freedom

	Value	Df	p-value
Pearson Chi-squared	34.319	15	0.003
Likelihood ratio	38.867	15	0.001
No. of valid cases	62		

Among all 62 cases included in this study, 45 cases were that of Squamous cell carcinoma (72.6%), nine cases were of CIN 1 (14.5%), three cases of CIN 2 (4.8%), two cases of CIN 3 (3.2%), and three cases of adenocarcinoma (4.8%) (Table 2).

**Table 2:** Distribution of patients according to histological diagnosis CIN - cervical intraepithelial neoplasia

Histologic type	No. of cases	Percentage
Squamous cell carcinoma	45	72.6
CIN 1	9	14.5
CIN 2	3	4.8
CIN 3	2	3.2
Adenocarcinoma	3	4.8
Total	62	100.0

**Figure 1:**
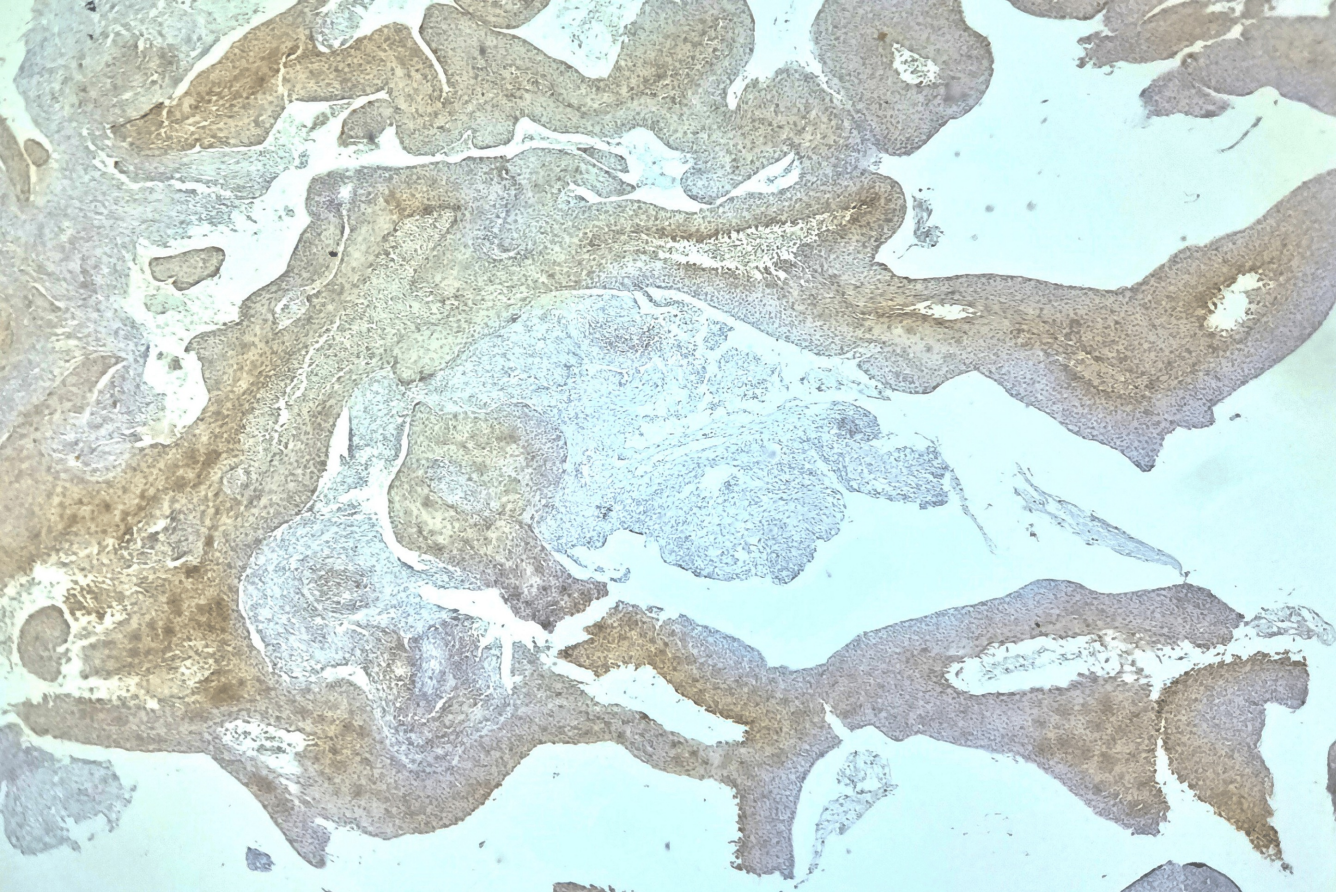
Photomicrograph showing 10-50% positive tumor cells, i.e., moderate expression of COX-2 (10x magnification)

**Figure 2:**
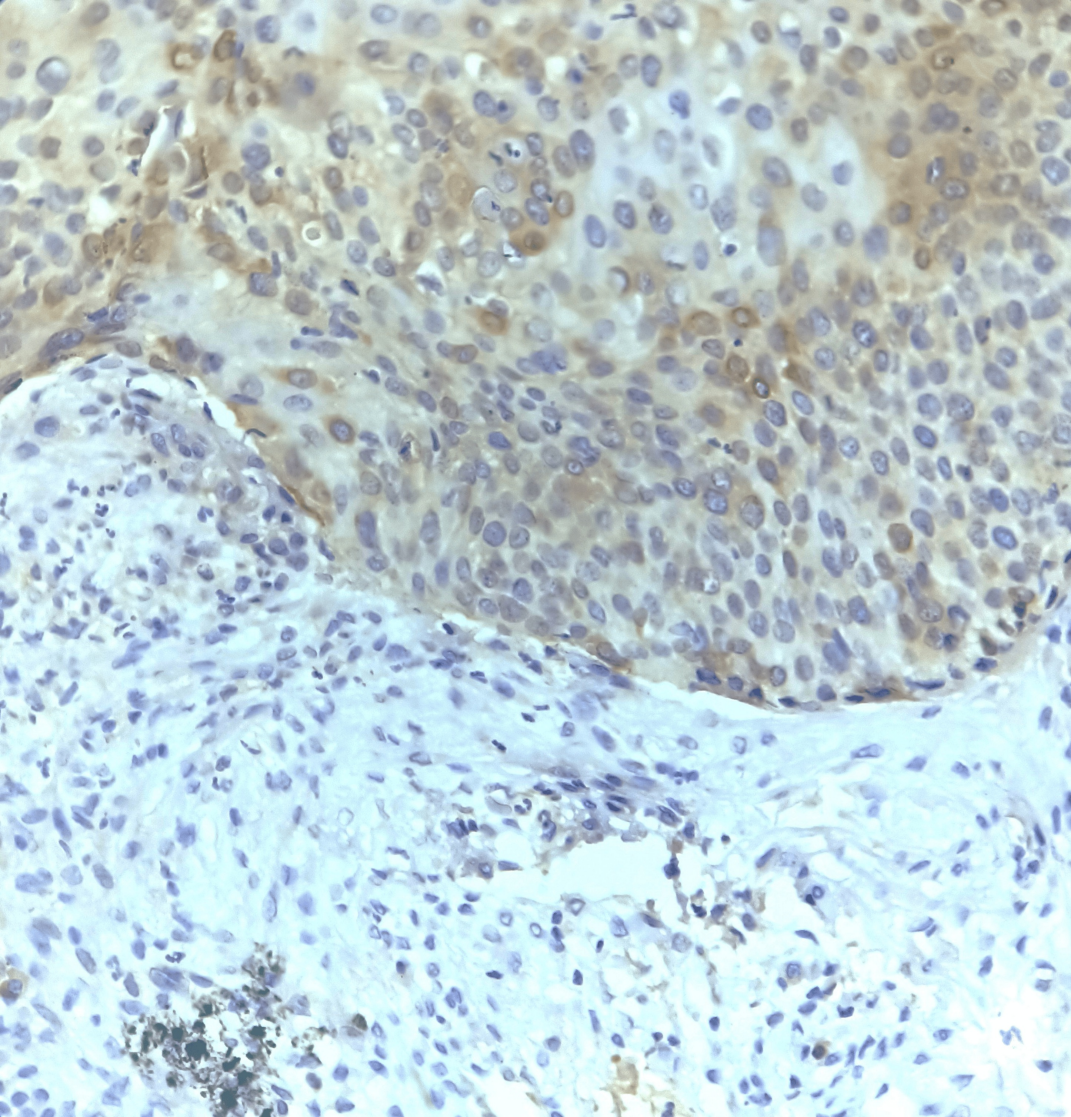
Photomicrograph showing 10-50% positivity of COX-2, i.e., moderate expression (40x magnification)

**Figure 3:**
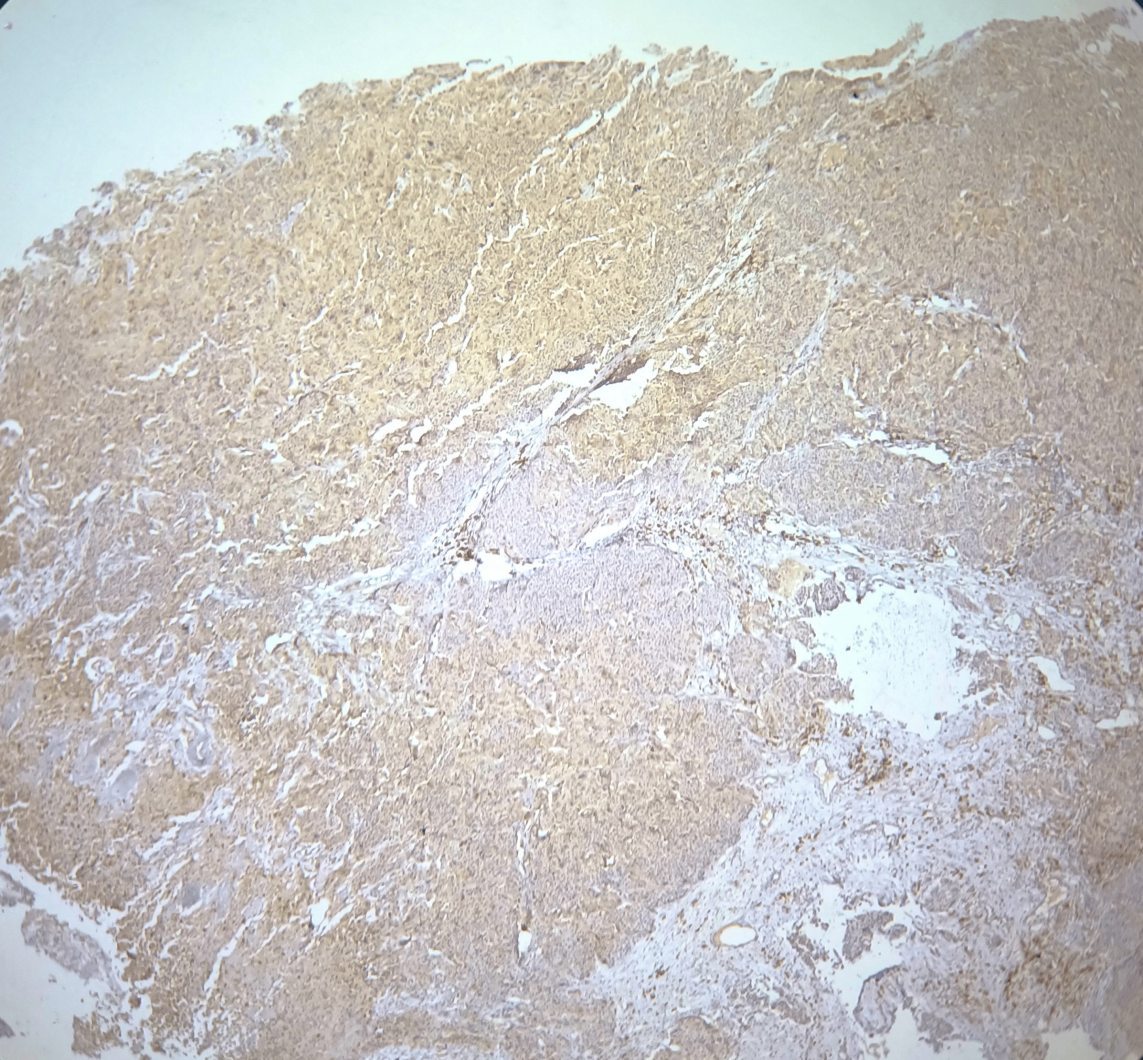
Photomicrograph showing >50% positive tumor cells, i.e., high expression of COX-2 (10x magnification)

**Figure 4:**
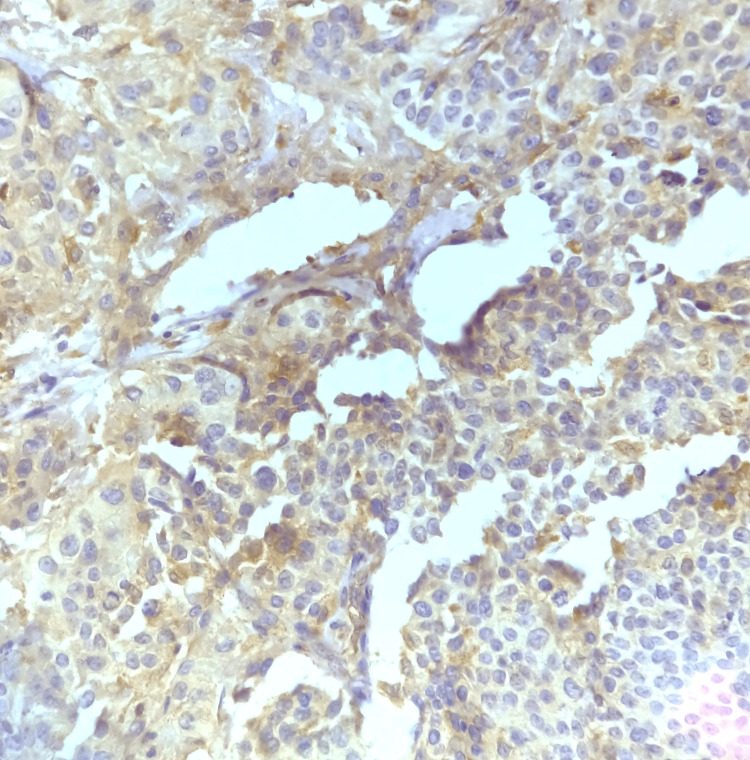
Photomicrograph showing >50% positive tumor cells, i.e., high expression of COX-2 (40x magnification)

Among all 62 cases included in this study, 45 cases were reported to be that of squamous cell carcinoma. Out of these 45 cases, 20 (32.3%) cases showed higher expression of COX-2 when compared with all cases (three out of nine cases showed low expression in cervical intraepithelial neoplasia (CIN) 1, two out of three cases showed low expression in CIN 2, two out of two cases showed moderate expression in CIN 3 and one out of three cases showed low expression in adenocarcinoma) as shown in Table 3, Figures 1-4.

**Table 3:** Expression of COX-2 with tumor differentiation *p-value: 0.003 (significant); SCC - squamous cell carcinoma; CIN - cervical intraepithelial neoplasia; ACC - adenocarcinoma

Histological grade	Undetected	Low expression	Moderate expression	High expression	Percentage	p-value
SCC	5 (8.1%)	7 (11.3%)	13 (21.0%)	20 (32.3%)	72.7	0.003*
CIN 1	6 (9.7%)	3 (4.8%)	0.0	0.0	14.5	
CIN 2	1 (1.6%)	2 (3.2%)	0.0	0.0	4.8	
CIN 3	0.0	0.0	2 (3.2%)	0.0	3.2	
ACC	2 (3.2%)	1 (1.6%)	0.0	0.0	4.8	

Upon statistical analysis, the p-value for the intensity of COX-2 staining with the tumor grade was found to be 0.003 (<0.05), therefore making the findings of this present study statistically significant.

## Discussion

Due to its probable involvement in the development and spread of tumors, cyclooxygenase (COX), the primary enzyme in prostaglandin metabolism, has been the subject of much research. The fact that tumor tissues have substantially greater prostaglandin concentrations than matching normal tissues is widely known. Both the early and late phases of carcinogenesis are significantly impacted by COX-2 expression. Due to the independent presence of COX-2 during the early phases of cell differentiation or replication, different malignant tumors frequently exhibit differences in COX-2 expression. In pre-neoplastic lung lesions, pre-invasive neoplasias of the breast, bladder, and pancreatic, as well as adenomatous and metaplastic lesions of the stomach, there is evidence of increased expression of COX-2. Despite its presence in invasive carcinomas, there are few investigations on COX-2 expression in cervical cancer [4].

Increased COX-2 expression has been linked to more severe dysplasia. According to the present study, there was a statistically significant (p<0.05) difference in COX-2 expression between invasive squamous cell carcinomas and in-situ carcinomas. Bandhyopadhyay et al. also observed a significant difference in COX-2 expression patterns between in-situ cervical intraepithelial neoplasia (CIN) and invasive squamous cell carcinoma (SCC), with higher expression in the invasive cases (p=0.0002) [4]. Balan et al. found that cytoplasmic COX-2 immunostaining intensity was weaker in specimens with low-grade squamous intraepithelial lesions (LSIL) and stronger in those diagnosed with high-grade squamous intraepithelial lesion (HSIL) [5].

In contrast to cervical intraepithelial lesions, we found that the intensity of COX-2 staining increased with the tumor grade in our investigation. Squamous cell carcinomas had the highest intensity of staining. At a p-value of 0.003, these results were statistically significant. Similar results were reported by Balan et al. and Fukazawa et al. [5,7]. However, Bandhyopadhay et al. did not find these results to be statistically significant [4]. Fukazawa et al. reported that the mean COX-2 expression increased significantly from CIN 1 to CIN 2, CIN 3, and SCC [7].

Cervical adenocarcinomas have been shown to express COX-2. In contrast to squamous cell carcinoma, cervical adenocarcinomas in the current study exhibited low positivity, with only one case out of three showing positivity. However, this variation was not found to be statistically significant. It is possible that the small number of cases limited the ability to draw definitive conclusions.

According to Manchana et al., COX-2 expression in cervical adenocarcinoma was higher than in squamous cell carcinoma [8]. Their study revealed a significant relationship between COX-2 expression and cervical adenocarcinoma. Baltazar et al. evaluated 130 cervical cancer patients for COX-2 and EGFR expression and found that adenocarcinomas (ACC) had higher levels of COX-2 expression than adenosquamous carcinomas (ASC) [9].

A study by Kim et al. concluded that patients who tested positive for COX-2 were the most suitable candidates for trials involving selective COX-2 inhibitor adjunctive therapy [3]. Another study by Khunamornpong et al. demonstrated that adenocarcinomas with strong COX-2 expression exhibited an unfavorable therapeutic response. [10].

In our study, out of 14 hysterectomy cases, no lymphovascular or parametrial invasion was noted. Therefore, we cannot comment on the correlation of COX-2 expression with lymphovascular and parametrial invasion. However, a study by Khunamornpong et al., with a sample size of 196 cases, found that lymphovascular space invasion was associated with COX-2 expression and lymph node metastasis in cervical squamous cell carcinoma (SCC) [10]. They concluded that COX-2 expression could facilitate lymph node metastases following lymphovascular space invasion. A similar study by Mandic et al. also showed a statistically significant correlation between COX-2 expression and the presence of lymphovascular invasion (LVI) [11].

These findings highlight the potential predictive value of COX-2 expression, which could be used in combination with other important variables to adjust postoperative adjuvant therapy in SCC [12-15].

## Conclusions

Immunohistochemistry profiles using multiple markers play a crucial role in diagnosing and understanding cervical carcinoma, a common tumor in women, and in designing patient therapy schedules. The increased expression of COX-2 in a number of cervical neoplasms, such as adenocarcinoma, cervical intraepithelial neoplasia (CIN), and squamous cell carcinoma (SCC), indicates that the protein may play a role in the development and spread of cervical carcinoma. Our research shows that the degree of cervical dysplasia increases with COX-2 expression intensity, moving from CIN to invasive malignancy. Unlike adenocarcinomas, cervical squamous cell carcinomas exhibited strong COX-2 positivity with intense staining. However, due to the absence of parametrial and lymphovascular invasion in 14 cases and the small sample size, we could not assess the correlation between COX-2 expression and these invasions. The limited sample size also resulted in fewer CIN and adenocarcinoma cases compared to SCC, which hindered our ability to draw definitive conclusions.
